# Modified percutaneous Kyphoplasty technique in the treatment of osteoporotic thoracolumbar burst fractures: could it reduce the odds of cement leakage?

**DOI:** 10.1186/s12893-020-00753-4

**Published:** 2020-05-07

**Authors:** Xuan-geng Deng, Xiao-ming Xiong, Dun Wan, Hua-gang Shi, Guo-long Mei, Wei Cui

**Affiliations:** Department of Spine, Sichuan Orthopedic Hospital, No.132, the west 1st section of Yihuan Road, Chengdu, 610041 China

**Keywords:** Percutaneous kyphoplasty, Osteoporosis, Compression fracture, Burst fractures

## Abstract

**Background:**

Osteoporotic thoracolumbar burst fracture (OTLBF) is common in seniors. Due to the fracture of the posterior vertebra and spinal canal occupancy, the risk of cement leakage and spine injury is high in OTLBF patients, thus the application of vertebroplasty and kyphoplasty is limited in these patients. This study aims to investigate the efficacy and safety of the modified percutaneous kyphoplasty (MPKP) in the treatment of OTLBF.

**Methods:**

Clinical data of the OTLBF patients treated with MPKP and the osteoporotic thoracolumbar compression fracture (OTLCF) patients undergone PKP from January 2014 to June 2016 were collected. The key procedure of the MPKP was to fill the bone cavity with gel-foam by the first balloon inflation and to press the gel-foam by a second balloon inflation. Pain intensity, Oswestry disability index (ODI), and bone cement leakage of the patients in the two groups were analyzed.

**Results:**

In the burst fracture group, the overall spinal canal occupancy was relatively low, and the maximum occupancy was 1/3 of the sagittal diameter of the spinal canal. The surgical duration was longer in the burst fracture group (39.0 ± 5.0 min with 95% CI: 37.7, 40.3) than in the compression fracture group (31.7 ± 4.3 min with 95% CI: 31.1, 32.3), and the difference between the two groups was statistically significant (*Z* = -8.668 and *P* = 0.000). Both the Oswestry disability index (ODI) and the visual analog scales (VAS) were apparently improved, but there was no significant difference between the two groups. Cement leakage occurred in 13 out of the 53 cases (24.5%) in the burst fracture group and 35 out of the 193 cases (18.1%) in the compression fracture group, and there was no significant difference between the two groups (*Z* = − 1.038 and *P* = 0.299). Neither group had consequential symptoms, such as spinal cord lesion, pain, and numbness of the peripheral nerve.

**Conclusion:**

Similar to the efficacy of PKP in the treatment of OTLCF, MPKP efficiently reduced the cement leakage rate and improved the safety of the surgery, although it prolonged the surgical duration and introduced more surgical steps.

## Background

Osteoporotic thoracolumbar fracture is a prevalent clinical disease among the elderly [1–4]. Vertebral augmentation (including PKP and PVP) is a safe, rapid, and simple treatment method, which can improve the support capability of vertebral body and reduce the possibility of secondary kyphosis. Thus, vertebral augmentation is often the first choice for the treatment of osteoporotic vertebral compression fracture (OVCF). Previous studies revealed that percutaneous kyphoplasty (PKP) had more advantages including better vertebral height restoration, better post-surgical function, and less bone cement leakage compared with percutaneous vertebroplasty (PVP), although the pain relief effect of these two methods was very similar [5–8]. Besides, compared with the patients without surgery or with PVP, the OVCF patients treated with PKP had the highest survival rates, lowest disability rates, and least complications despite the relatively high cost during early treatment [9].

A multi-center study indicated that approximately 50% of the OTLF cases had burst fractures [10]. Patients with burst fractures have a higher rate of intraspinal cement leakage because the rupture of the posterior wall of the vertebral body leads to the loss of a natural barrier against intraspinal cement leakage during vertebral augmentation. Thus, compared with compression fracture, the greatest risk of burst fracture is nerve or spinal cord injury due to cement leakage. Studies have shown that neither PKP nor PVP can restore the mechanical function of the spine with burst fractures [11, 12], and considering the safety of the surgery, burst fracture is a contraindication or a relative contraindication of vertebral augmentation [13]. Therefore, currently, the most frequently used surgical procedures are vertebral internal fixation and anterior support, such as titanium mesh cages [14, 15]. However, in senior patients, due to old age and osteoporosis, the use of internal fixation for fracture treatment is limited, and major surgeries might result in higher death rates [13].

From the perspective of surgical procedure, PKP is still the least invasive and most efficient treatment for pain relief, and it is effective for the treatment of burst fractures with painful anesthesia that can instantly improve the early stability of OTLBF [16–19]. Therefore, it would be a great benefit for patients if the risk of intraspinal bone cement leakage could be reduced, or the safety of the surgery could be similar to that of compression fracture. However, there were limited studies on how to ensure surgical safety and improve the procedure at a low cost. This study was, therefore, designed to reduce the intraspinal bone cement leakage and the risk of nerve or spinal cord injury by improving the percutaneous kyphoplasty procedure for OTLBF.

## Methods

### Clinical cases

In this study, we collected and analyzed cases with single vertebral osteoporotic fracture treated from January 2014 to June 2016. Cases of burst fractures treated with modified percutaneous kyphoplasty (MPKP) and cases of compression fractures treated with PKP were included in this study. All cases met the following criteria: no signs or symptoms of a nerve injury, BMD T-score was ≦ − 2.5SD by bone density test (dual-energy X-ray absorptiometry, Hologic USA). All burst fracture cases were diagnosed as the A3 type based on the AO spine classification [20], and their scores were lower than 4 according to the Thoracolumbar Injury Classification and Severity Score (TLICS) system [21]. Five of the 53 patients with burst fracture had surgery at least 2 weeks after the injury, whereas 48 patients had surgery within 2 weeks of the injury. The average age of the patients was 69.9 ± 6.7 years (95% confidence interval: [68.2, 72.0]). The average follow-up time after surgery was 12.1 ± 4.2 months (95% CI: [10.9, 13.2]).

The patients with compression fracture were A1 type according to the AO spine classification [20]. Out of the 193 compression fracture cases (52 males and 141 females), 38 had the surgery at least 2 weeks after the injury, whereas 155 cases had the surgery within 2 weeks of the injury. The average age of the patients was 71.3 ± 5.7 years (95% CI: [70.3, 72.0]). The average postoperative follow-up time was 11.7 ± 4.4 months (95% CI: [11.2, 12.5]).

### Preparation before the surgery

Imaging examinations included X-ray examination, magnetic resonance imaging (MRI), and computed tomography (CT) of the injured spine and the peripheral regions. The goal of the imaging examinations was to evaluate the dural sac and nerve roots and to modify the surgical procedure by investigating the fracture line, the morphology, and the size of the bone block protruding into the spinal canal.

### Surgery method

In the current study, MPKP was used to treat OTLBF. MPKP has the following advantages compared with PKP. 1). Gel-foam slices were pushed into the bone cleft by the first balloon inflation, 2). Second balloon inflation was applied to tamp the gel-foam slices, 3). Bone cement was injected with low resistance (pushing-stop technique).

All patients took a prone position, received general anesthesia, and were electrophysiologically monitored. C-arm fluoroscopy was performed to locate the biopsy site. The surgical puncture was performed following the previously reported method [22]. Briefly, under C-arm monitoring, two trocar needles were used for the puncture, which avoided the fracture line and the posterior region of the displaced bone block protruding into the spinal canal. The inclination angle of the needles was small to avoid pushing the fractured bone block into the spinal canal during balloon inflation.

**Fig. 1:**
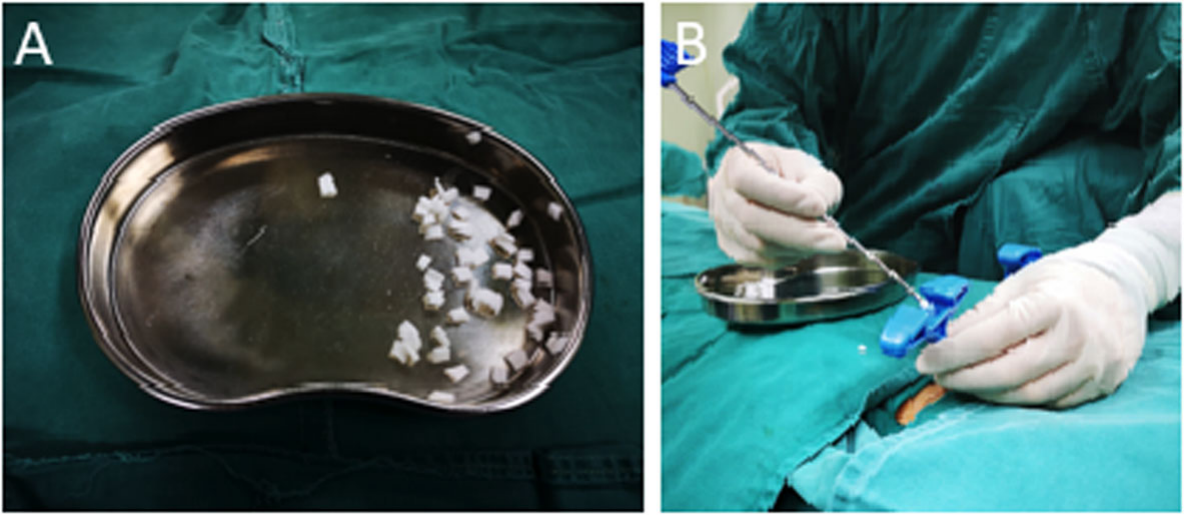
Gel-foam slice and injection. Panel **a:** Bone cement was sliced into pieces with a size of 0.5 × 0.5 cm. Panel **b**: Injection of sliced gel-foam into the bone cavity through puncture cannula for balloon inflation

One piece of gel-foam was cut into fine slices (Fig. 1a), and these slices were pushed into the bone cavity by the first balloon inflation through bilateral channels (Fig. 1b). Then, the balloon was inserted and inflated for a second time. Bone cement was injected when it was at the toothpaste-like phase. The injection was performed under X-ray monitoring and at a low-resistance condition using a push-stop alteration technique. The surgery was ended when the bone cement reached the target zone at the posterior 1/3–1/4 of the vertebral body, or when the fragments of the posterior bone were glued to the vertebra by bone cement so that the anterior and middle vertebrae were stabilized, or when the suspected intraspinal cement leakage occurred by monitoring the electrophysiological signal and nerve functions. Immediate decompression of the spinal canal was prepared if any corresponding symptoms appeared, but it did not happen in the current study.

The patients could ambulate with support 2 h after the surgery, and the patients were re-examined by X-ray. If cement leakage was suspected, the patients were further examined by CT scan.

### Comparison

Visual analog scale (VAS) and Oswestry Disability Index (ODI) were compared before the surgery, on the day after the surgery, and at the last follow-up. The ODI omitted the questions about sexual function due to Chinese culture. The bone cement leakage rate was determined and analyzed by X-ray and confirmed by CT. When the bone cement coverage was bigger than the vertebral region, it was considered as cement leakage. The percentage of ODI was calculated as following: ODI = (Actual index / 45) × 100%.

### Statistical method

The SPSS 18.0 software (IBM, US) was used for statistical analysis. The test level *α* of 0.8 and the *P*-value < 0.05 were considered statistically significant. VAS, ODI, and surgery duration were represented as mean ± standard deviation (SD). The leakage rate was determined in two independent samples by non-parametric test. The parametric and normally distributed VAS, ODI and surgery duration were analyzed by *t*-test for two independent samples. Non-parametric tests for two independent samples (Mann-Whitney U) were used to analyze data that were not normally distributed. Comparisons within groups were conducted using non-parametric test (Wilcoxon) for two related samples.

## Results

There were no statistical differences in age (*Z* = − 1.296 and *P* = 0.195) and gender (*Z* = − 0.735 and *P* = 0.462) between the two fracture groups. In the burst fracture group, the bone occupancy was 1/3 of the sagittal diameter of spinal canal in the majority of the cases. The average surgery duration was 39.0 ± 5.0 min (95% CI: [37.7, 40.3]) for patients with burst fracture and 31.7 ± 4.3 min (95% CI: [31.1, 32.3]) patients with compression fracture, and there was a significant difference between the two groups (*Z* = -8.668 and *P* = 0.000). As summarized in Tables 1, 2, and 3, bone cement leakage (no spinal canal cement leakage) occurred in 13 out of the 53 cases (24.5%) in the burst fracture group and 35 out of the 193 cases (18.1%) in the compression fracture group, and there was no significant difference between the two groups (*Z* = − 1.038 and *P* = 0.299). Moreover, there were no postoperative symptoms, such as spinal cord lesion, pain, or numbness of the peripheral nerve, in either of the groups. The zones where the leakage occurred were listed in Table 1. Below, we describe two representative cases to explain in detail the MPKP treatment of OTLBF.

**Table 1:** Summary of bone cement leakage in both OTLBF group and OTLCF groups

Leakage	Canal	Foramen	Around Vet.	Inter disc	Non
OTLBF (*n* = 53)	0	0	11	2	40
OTLCF (*n* = 193)	1	0	29	5	158

*OTLBF* Osteoporotic thoracolumbar burst fractures; *OTLCF* Osteoporotic thoracolumbar compression fractures

**Table 2:** Comparison of pain relieve during the treatment

Items	Pre-VAS	Pro-VAS	Value of Stat.	Last-VAS
OTLBF (n = 53)	7.33 ± 0.95	2.42 ± 0.79	*Z* = -6.4013 *P* = 0.000	1.50 ± 0.58
OTLCF (*n* = 193)	7.16 ± 0.77	2.26 ± 0.71	*Z* = -12.220 *P* = 0.000	1.51 ± 0.62
Value	*Z* = -1.245 *P* = 0.213	Z = -1.514 *P* = 0.130		*Z* = -0.052 *P* = 0.959

*OTLBF* Osteoporotic thoracolumbar burst fractures; *OTLCF* Osteoporotic thoracolumbar compression fractures

**Table 3:** Comparison of ODI change during the treatment

Items	Pre-ODI	Last-ODI	Value of Stat.
OTLBF (*n* = 53)	0.73 ± 0.07	0.23 ± 0.06	*Z* = -6.336 *P* = 0.000
OTLCF (*n* = 193)	0.72 ± 0.07	0.22 ± 0.06	*Z* = -12.050 *P* = 0.000
Value	*Z* = -0.918 *P* = 0.359	*Z* = -1.157 *P* = 0.247	

*OTLBF* Osteoporotic thoracolumbar burst fractures; *OTLCF* Osteoporotic thoracolumbar compression fractures

**Fig. 2:**
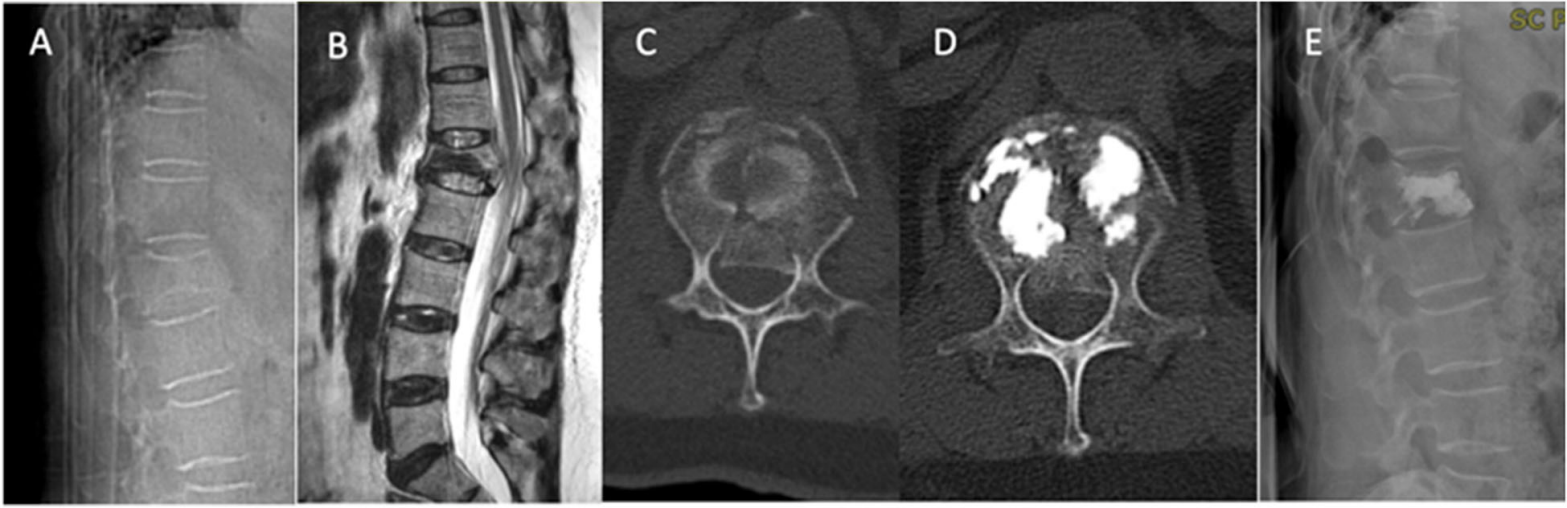
Images of patient #1. **a**: Preoperative X-ray radiograph of L1 fractures at the standing position; **b**: preoperative weighted MRI image of T2, **c**: axial CT image of L1; **d**: postoperative CT image shows the fracture area filled with cement. **e**: Postoperative X-ray radiographs, no cement leakage into the spinal canal

*Patient 1*: male, 82 years old, admitted 1 month after an accidental falling, he had back pain without nerve injury symptoms; the VAS score was 6 and the ODI score was 7. Figure **2**a shows a preoperative X-ray radiograph, which indicates an old fracture at L1 (Fig. 2a). Figure 2b is a preoperative MRI image of T2, which shows a burst fracture at L1 and the partially occupied spinal canal by extrudate. Figure 2c is the CT scan of L1, which indicates an old fracture at L1 with occupancy in the spinal canal. Figure 2d and e show the postoperative X-ray radiographs, which clearly show that the height of the vertebral body L1 has been restored and that the cement is evenly distributed up to the posterior 1/3 of the vertebral body without cement leakage. The VAS score is 2.

**Fig. 3:**
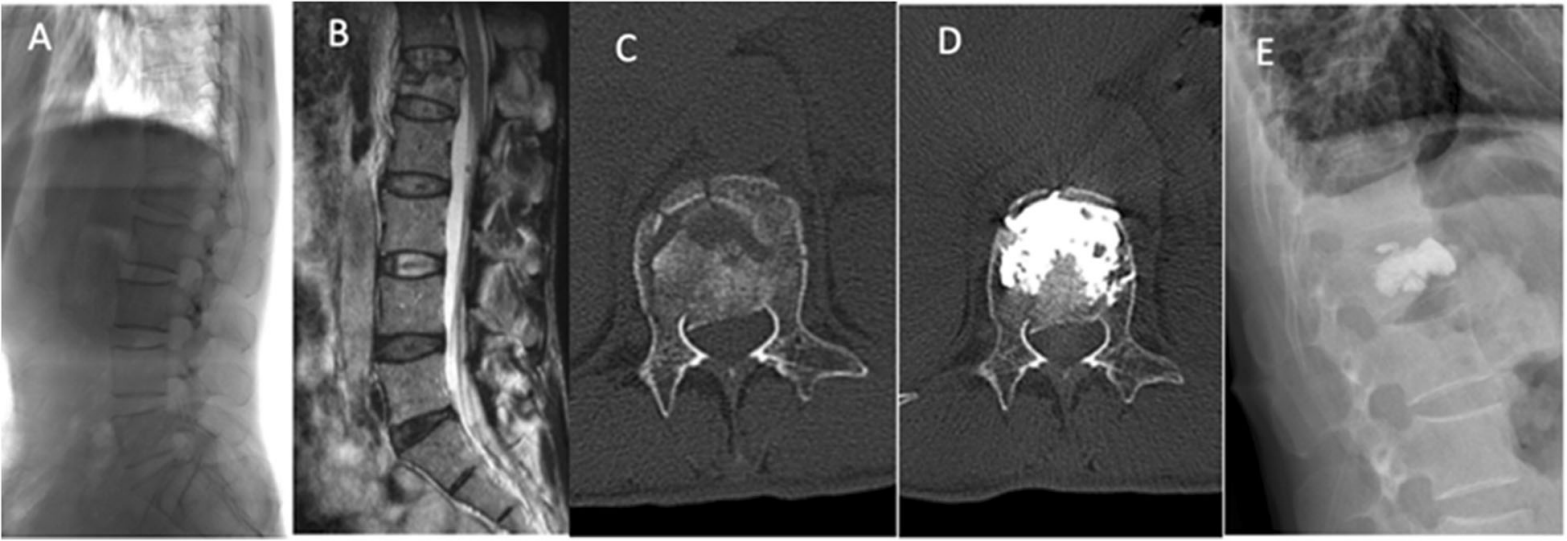
Images of patient #2. **a**: X-ray radiographs before surgery; **b**: preoperative weighted MRI image of T2; **c**: axial CT image of L1; **d**: postoperative CT image shows that most of the fracture line is filled with cement without leakage; **e**: postoperative X-ray shows that the fracture line is filled with cement without leakage

*Patient 2*: female, 60 years old, admitted 5 days after falling, the VAS score was 8, and ODI was 7.5. Figure 3a shows the preoperative X-ray radiograph, which shows a burst fracture at L1 and the wedge deformities of the vertebral body. Figure 3b is a preoperative MRI image of T2, which exhibits a burst fracture of L1 and an occupancy in the spinal canal at L1. Figure 3c shows the axial CT image of L1, where a fracture is found at the posterior border of the vertebral body, and the bone block occupies 1/3 of the sagittal diameter of the spinal canal. Figure 3d is the postoperative axial CT image of L1. As shown in the figure, the cement is evenly distributed inside the fracture line without leakage. Figure 3e is an X-ray radiograph at 22 months after the surgery, which displays evenly distributed cement with mild leakage at the vertebral superior endplate.

## Discussion

Although most OTLBF cases can be treated noninvasively, patients who have intractable pain or patients who cannot tolerate pain at the early stage require surgical intervention. Although PKP is a micro-invasive method, due to the prohibition by law and IRB policy, PKP was not used for OTLBF treatment in the current study. However, PKP was used for the treatment of compression fracture in the current study, which served as a control for the analysis of the outcome of MPKP treatment of OTLBF.

### Preoperative planning and puncture

The major risk of PKP treatment for burst fracture is the leakage of bone cement as well as the displacement of the fractured block to the spinal canal, which compresses nerve and spinal cord. Therefore, it is critical to reduce the risk of cement leakage to the spinal canal during PKP. The main route of leakage is along the fracture line, whereas the minor one is the venous sinus in the middle of the vertebral body. Keep this in mind, to reduce the risk of cement leakage, not only should the puncture needle trajectory avoid the fracture lines identified by CT or MRI, but also the expansion of the fracture lines be avoided when the balloon is inflated. The major fracture line gets “sealed” via the inflation of the balloon.

No differences in the effectiveness of the treatment of osteoporotic compression fracture (OCF) by either unilateral or bilateral injection were found in earlier studies [23]. The unilateral kyphoplasty has advantages in terms of shorter surgery time, lower radiation and bone cement dose, and reduced risk of cement leakage [24, 25]. However, there is a potential risk that the inflation of the balloon will push the bone block of the posterior wall further into the spinal canal if the balloon stays at or near the center of the vertebral body, and there is a higher risk of nerve injury since unilateral injection normally has large insertion and inclination angles. In contrast, the bilateral injection has a relatively smaller insertion angle and has a relatively smaller squeezing effect on the posterior wall during balloon inflation.

### Balloon inflation and gel-foam filling

The most likely path for cement leakage is the fracture line and the intervertebral space since these regions are the least resistant to the diffusion of cement. Among the patients with fractures older than 2 to 3 weeks, the fracture line is partially sealed by fibrous tissues, bone matrix, or new bone [26], and thus the risk of bone cement leakage is reduced in these cases. This could be the reason that less cement leakage occurred in the cases with old fractures in the compression fracture group, and no leakage was detected in the burst fracture patients with old fractures in our study. The balloon can be used to squeeze and seal the previous fracture line through the inflation of the balloon. Furthermore, we attempted to fill the fibrous tissue-containing fracture line and cavity with gel-foam to prevent cement leakage by using a second balloon inflation, which was described in a previous report, to push the gel-foam into the low-resistance zones of the vertebral body and to seal the regions with a potential high risk of leakage [27]. However, it was also reported that gel-foam did not reduce cement leakage in vertebroplasty [28]. The methods of gel-foam injection were identical in these two studies. The gel-foam was first cut into fine pieces and then mixed with a liquid for injection, which is different from the method used in our study. Although the gel-foam showed advantages of good biocompatibility and low price in previous studies, it can be replaced by newly developed material. Also, large scale randomized clinical studies are required to further determine the clinical outcome of gel-foam filling in PKP or MPKP.

The anterior vertebral height should be restored and enhanced during PKP or MPKP because the compression of the anterior vertebral body, the fracture of the posterior wall, the occupancy in the spinal canal, and the local kyphosis of the spine may lead to displacement of the posterior fractured block to the spinal canal at standing or walking posture. Previous studies on the clinical treatment of compression fractures found that PKP was able to restore the vertebral height [5–8], which was also confirmed in the current study, that is, the vertebral height was partially restored by the surgery. In the current study, no matter it is the result of postural reduction or balloon inflation, the restoration of the anterior vertebral height and the adhesive effect of bone cement not only reduced the kyphotic angle of the fractured vertebral body at the posterior fracture site but also decreased the potential risk of the displacement of the posterior fractured block into the spinal canal. Although limited vertebral height restoration has been reported in previous studies [8, 29], the stabilization effect of bone cement has not been extensively studied and remains to be further studied. In addition, there is a difference in the bilateral injection used in the current study and that used by other investigators. We inflated the same balloon sequentially for bilateral injection, whereas other investigators inflated the balloons simultaneously for the injection [30].

### Bone cement injection

The positive effects of vertebral augmentation are the stabilization of the fractured region by bone cement, the prevention of the subtle movements of fractured trabeculae and the resulting pain, and providing a stable environment for bone healing [31]. Since there is a fracture at the posterior 1/3 of the vertebral body in burst fracture, bone cement needs to diffuse to the posterior 1/3 of the region to stabilize the posterior fracture. Therefore, another key favorable role of vertebral augmentation is to prevent the displacement of the posterior fractured block by stabilizing the anterior part of the vertebral body or the posterior fractured block through the adhesive effect of bone cement (as in the cases 1 and 2 of the current study).

In our opinions, other key factors influencing the leakage include the time point of cement injection and the injection method used. In terms of the time of cement injection, we recommend the injection to be done when the cement is in the toothpaste-like phase because the risk of leakage is high when the cement is still in the liquid phase. At the beginning of injection, sometimes the bone cement diffuses rapidly to or near the posterior wall of the vertebral body because there is a relatively large fracture line leading to the spinal canal. In this case, we stopped the injection immediately to allow the cement to cure. Then the injection was resumed until the fracture was completely sealed. This is similar to the procedure described by Wu et al [32] A similar surgical operation was performed in Yang’s study, in which a balloon was used to create a cement cavity, and cement injection was resumed when the previously injected cement was cured [33]. Most importantly, the slow and intermittent injection is crucial to lower the diffusing pressure of cement, which in turn reduces the risk of leakage.

Bone cement is diffused in the vertebral body according to the gradient of resistance, and in general, it fills the spaces between the bones and the trabeculae along the fracture line, where the low-resistance regions are located. In burst fracture, the regions where cement diffusion occurs most easily are the fracture line and the cavity formed after a fracture. Hence, the use of gel-foam or cement filling can theoretically reduce the possibility of cement leakage in these regions.

To stabilize the posterior vertebral body and reduce the resistance against the injection during the late period of cement injection, we gradually pulled the needle out to the middle or posterior 1/3 of the vertebral body while injecting the bone cement. The reason for pulling out the needle is to facilitate the diffusion of cement to the posterior vertebral body as much as possible under low resistance. Nevertheless, the most important goal is to prevent cement leakage into the spinal canal through strict monitoring. Currently, to the best of our knowledge, there is no study about the correlation between the distribution range of bone cement and the vertebral stability and clinical outcomes. Empirically, in burst fracture complicated with middle column injury, only cement diffusion in the posterior vertebral body can stabilize the vertebral body and relieve the pain.

In some studies, a container was used to effectively reduce the cement leakage [34, 35]. Most of the cement was restrained inside the container, and a limited amount of cement leaked out of the container and anchored to the surrounding bone tissue through the adhesive effect. However, the feasibility of preventing cement leakage by using container in burst fractures remains to be further tested and evaluated in clinical studies.

### Surgery outcomes

After surgery, the pain was significantly relieved in the burst fracture group, which was similar to that in the compression fracture group. In addition, ODI value indicated remarkable improvement in function recovery in OVTLF patients, suggesting that MPKP is a good option for OTLBF patients.

The surgery duration of MPKP was longer than that of PKP treatment for compression fracture. The prolongation of time can usually be explained from the following three aspects: the use of a bilateral injection, the filling of the fracture line with gel-foam and the second inflation of the balloon, and the two-step cement injection procedure.

It has been previously reported that bone cement leakage rate in the PKP treatment of OTLBF was 23.1–45.4% [36, 37], and the leakage rate of MPKP in the current study was 24.5%, suggesting that the outcome of the MPKP treatment of OTLBF in the current study is acceptable. Cement leakage occurs around the vertebra and in the intervertebral space, which does not cause symptoms in general. Furthermore, in the present study, the cement leakage rate of MPKP treatment of OTLCF was not statistically different from that of PKP treatment. More importantly, there was no risk of cement leakage into the spinal canal in the patients treated with MPKP. These findings suggested that MPKP can effectively reduce bone cement leakage rate and improve the safety of the surgery despite longer surgical time and slightly more complicated procedure.

The major limitation of the current study is that it is a single-center study with a limited number of cases. Besides, the cases of spinal canal occupancy were mild, and the control group was OTLCF treated with PKP, which may weaken the reliability of the conclusions.

## Conclusions

MPKP can efficiently reduce the rate of cement leakage into the spinal canal, partially restore vertebral height, and improve the safety of the surgical procedure, although the duration of the surgery is prolonged and the surgical procedure is more complicated.

## Data Availability

The datasets used during the current study available from the corresponding author on reasonable request.

## Abbreviations

MPKP: Modified percutaneous kyphoplasty
OTLBF: Osteoporotic thoracolumbar burst fracture
OTLCF: Osteoporotic thoracolumbar compression fracture
ODI: Oswestry disability index
PKP: Percutaneous kyphoplasty
PVP: Percutaneous vertebroplasty
